# Age – specific incidence rate change at breast Cancer and its different histopathologic subtypes in Iran and Western countries

**Published:** 2013

**Authors:** Maryam Sadat Hosseini, Maliheh Arab, Behzad Nemati Honar, Giti Noghabaei, Nazanin Safaei, Tahereh Ghasemi, Farah Farzaneh, Tahereh Ashraf Ganjoie

**Affiliations:** 1 Maryam Sadat Hosseini, Maliheh Arab, Professor of Gyneco-Oncology, Preventative Gynecology Research Center (PGRC), Imam Hossein Medical Center, Shahid Beheshti University of Medical Sciences, Tehran, Iran.; 2 Behzad Nemati Honar, Assistant Professor of General Surgery. Preventative Gynecology Research Center (PGRC), Imam Hossein Medical Center, Shahid Beheshti University of Medical Sciences, Tehran, Iran.; 3 Giti Noghabaei, General Physician, Imam Hossein Medical Center, Shahid Beheshti University of Medical Sciences, Tehran, Iran.; 4 Nazanin Safaei_, _Resident of Obstetrics and Gynecology, Preventative Gynecology Research Center (PGRC), Imam Hossein Medical Center, Shahid Beheshti University of Medical Sciences, Tehran, Iran.; 5 Tahereh Ghasemi, Resident of Obstetrics and Gynecology, Preventative Gynecology Research Center (PGRC), Imam Hossein Medical Center, Shahid Beheshti University of Medical Sciences, Tehran, Iran.; 6 Farah Farzaneh, Associate Professor of Gyneco-Oncology, Preventative Gynecology Research Center (PGRC), Imam Hossein Medical Center, Shahid Beheshti University of Medical Sciences, Tehran, Iran.; 7 Tahereh Ashraf Ganjoie, Associate Professor of Gyneco-Oncology, Imam Hossein Medical Center, Shahid Beheshti University of Medical Sciences, Tehran, Iran.

**Keywords:** Age groups, Breast cancer, Histopathology, Incidence

## Abstract

***Objective:*** The aim of the present study was to determine the frequency and age-specific incidence rate of different histopathologic subtypes of breast cancer in Iran, and compare it to neighboring and Western countries and to discuss the probable effective main factors.

***Methods:*** National data from cancer registry for 6265 female breast cancer patients were studied in 10 histopathologic groups.

***Results:*** The most common tumor was ductal carcinoma (89%). The peak age – specific incidence rate of breast cancer in total, and for epithelial, non-epithelial and ductal carcinomas were all 50-59 years, and it decreased in older age. It is in contrast to US SEER report which shows the incidence increases in higher age.

***Conclusion:*** Three main factors including younger age of Iranian patients, probable more ERN tumors and different histopathological profile of breast cancer in Iran might be considered and studied to explain different slope of breast cancer after menopause compared to other countries.

## INTRODUCTION

Breast cancer is the most common cancer among females in the world.^1^ Age-specific incidence of breast cancer is not similar in different regions. In Asia the maximum incidence is in 40 – 50 age groups. In Contrast, in western countries the increase in incidence continues as the age increases.^2^

The frequency of the most common histopathology of breast cancer, ductal carcinoma, has been reported to be about 70% in US SEER (1999 -2001), 81.8% in Jordan, 84.7% in Egypt and 80.7% in Cyprus.^3^

The age-specific incidence rate of breast cancer shows three patterns. The first pattern is a rapid increase until menopause, followed by slow increase. The second pattern is rapid increase until menopause followed by platue and the third pattern is steady increase by age independent of menopause.^4^

Some histopathologic subtypes of breast cancer such as medullary and inflammatory subtypes are mostly poorly differentiated with low estrogen receptor content.^4^ Many studies have suggested breast cancer in younger age to be more aggressive and more likely to be estrogen receptor negative (ERN).^5^^-^^8^

The present study was conducted to determine the frequency and age – specific incidence rate pattern of different histopathologic subtypes of breast cancer in Iran, from 2005 to 2006 and compare different incidence rate change patterns with Iran’s neighboring countries and western countries and finally discuss probable main factors.

## METHODS

National data from formal cancer registry report of the Iranian Ministry of Health Treatment and Medical Education from 2005 to 2006 including 6674 breast cancer cases (ICD–0–2/3) were reviewed. Among these cases, 196 pre-invasive and 213 male breast cancers were excluded with 6265 cases remaining in the study.

Based on the world Health organization (WHO) histopathologic classification of breast cancer, tumors were studied in 6 groups including epithelial, mesenchymal, malignant lymphoma, fibroepithelial, nipple and metastatic. Pathological reports impossible to classify in this system, were grouped based on No.42 report of IARC^9^ in four other classes including: not site–specific carcinoma, other, not site–specific sarcoma and accepted with any site code. In this way ten groups are reported. In the next step, age–specific incidence rate of breast cancer and different histopathologic subtypes were reported in 9 age groups.

Description and data analysis statistical methods for frequency and incidence rates were made using Microsoft office excel 2007. Ethics committee of Gynecology Translational Research Center approved the study.

## RESULTS

Among 6265 studied breast cancer patients, the most common form was epithelial tumors including 5671 cases (90.5%). Table-1 shows the frequency of different histopathologies.

**Table-1:** The frequency of different histopathologies of breast cancer in Iran, 2005-6

*Histopathology*	*Number (Percent) *
Epithelial	5671 (90.52)
Mesenchymal	3(0.05)
Fibroepithelial	19 (0.30)
Malignant lymphoma	16(0.26)
Malignant paget’s	49 (0.78)
Metastatic	12 (0.19)
Other cancers	234 (3.74)
Not site – specific carcinoma	220 (3.51)
Not site – specific sarcoma	6 (0.10)
Any site	35(0.56)
Total	6265 (100)

The most common epithelial histopathology was ductal carcinoma, including 5037 cases (89%) (Table-II).

**Table-II:** Frequency of different epithelial breast cancer histopathologies in Iran, 2005-6

***Type of hystopathology***	***N (%)***
**Ductal carcinoma**	5037(89)
**Lobular carcinoma**	340(6)
**Medullary carcinoma**	170(3)
**Mucinous carcinoma**	56(1)
**Others**	56(1)
**Total**	5671(100)

Median age of total breast cancer cases and epithelial cases was 49 years and in non-epithelial tumors it was 52 years. Maximum age-specific incidence rate of breast cancer was 75.92 per 100.000 in 50-59 age group and decreased thereafter. Maximum age-specific incidence rate of epithelial tumors and its most common subtype, that is ductal carcinoma as well as non-epithelial tumors all were in the same age group 50-59, and all decreased in older ages (Table-3). Just in malignant paget’s, age-specific incidence rate continued to increase with age.

**Table-III:** Age – specific incidence of breast cancer and some common histopathologic subtypes in Iran, 2005-6

*Age group*	*All breast cancer*	*All epithelial tumors*	*Invasive ductal carcinoma*	*Malignant Paget*	*Not site-specific carcinoma*	*Other breast carcinoma*
20-29	2.66	2.24	1.90	0.02	0.06	0.13
30-39	24.97	22.77	20.93	0.07	0.94	0.85
40-49	62.23	56.98	50.24	0.37	1.87	2.43
50-59	75.92	68.60	60.14	0.59	3.26	2.92
60-69	66.07	59.90	52.84	0.89	2.27	2.11
70-79	48.17	43.47	38.89	1.11	0.99	2.11
80 +	48.45	41.67	36.34	0	3.88	2.91

**Fig.1:**
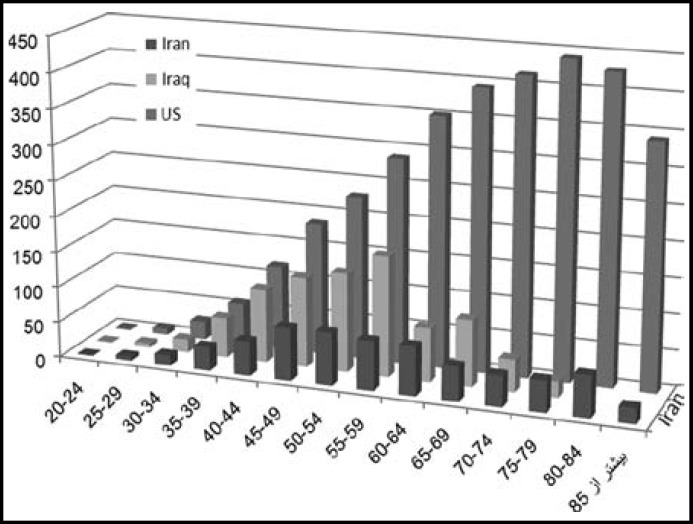
Comparison of age–specific incidence rate of breast cancer in Iran, Iraq and US

Present data of age-specific incidence rate of breast cancer in Iran, 2005-6 in comparison to Kordish Sulaimaniyah located in Iraq, the neighboring country to Iran, 2006-8^10^ and US SEER 2002-6^11^^,^^12^ are presented in Fig.1.

## DISCUSSION

The present study revealed epithelial tumors (90%) as the most common histopathology among 6265 breast cancer cases in Iran, in 2005-6. Among epithelial tumors, invasive ductal carcinoma (80% of total cases) and lobular carcinoma (5.5%) were the most common subgroups respectively, including 85.5% of the total counts. 

American National cancer institute's surveillance, epidemiology, and End Results (SEER) program reported 189634 female breast cancer cases during 1992-9. The most common histopathology being ductal carcinoma (73.7%) and lobular carcinoma (8.4%), including 82.1% of the total counts.^4^

Histopathologic features of 50 breast cancer cases in Rawalpindi, Pakistan, 2008, showed 84% invasive ductal carcinoma and 12% lobular carcinomas, including 96% of total.^13^Invasive ductal carcinoma is the most common histopathology of breast cancer following by lobular carcinoma and this pattern seems to be similar in Iran and US.

The age-specific breast cancer is increasing up to 50- 54 age group in Iran, and 55-59 age group in Iraq followed by a reduction in older age while an steady increasing pattern in all ages happens in the US (Fig.1).

***Different Histopathology: ***In SEER study^4^ histopathologic subtypes of ductal, lobular, medullary, inflammatory, papillary and mucinous breast carcinoma were studied regarding the pattern of incidence change by age. Ductal and lobular carcinoma as the most common histopathologic subtypes revealed a similar pattern with total cases, that continues to increase after menopause age (about 52) with slower slope. Medullary and inflammatory subtypes failed to increase after 52 years and revealed platue. Papillary and mucinous subtypes continued to increase in incidence steadily with the same slope by increasing age.

In the present study histopathologic subtypes might influence the overall pattern. However, medullary, inflammatory, papillary and mucinons subtypes' are unusual and data of present study included just one year (2005-6), so comparison of the pattern change of age-specific incidence rate for these subtypes was not possible.

***Different estrogen receptor status: ***In the SEER study^4^ estrogen receptor status had a prominent effect in age- specific incidence pattern. That is, negative estrogen receptor (ERN) cases of breast cancer did not reveal age- specific increase after 52 years old. In total cases as well as ductal carcinoma with ERN platue of increase was observed after 52 years.

In lobular subtype the increase of incidence continued alter 52 years, although among, medullary and inflammatory subtypes with ERN a significant decreased incidence was observed. 

Papillary and mucinous subtypes continued to increase in incidence with steady slope by aging, irrespective of estrogen receptor status. Another study confirmed the effect of ER, indicating that if positive, the risk continuous after menopause and if negative the risk clearly diminishes.^14^

In the present study estrogen receptor status is not known. Decreased age-specific incidence of breast cancer in Iran after menopause just similar to ERN cases might be the effect of probable higher number of ERN cases of breast cancer in Iran.

***Different tumor biology in younger age: ***Young breast cancer patients are more likely ERN^5^^-^^8^, and with a higher chance of harboring aggressive histopathologies.^15^ Median age of breast cancer in the present study was 49, showing a young population. The peak age of the breast cancer was between 40 and 50 years in Asian countries, whereas the peak age in the Western countries was between 60 and 70 years.^16^

## CONCLUSION

The age- specific incidence rate of breast cancer decreases after menopause in Iran which is in contrast to US, where it continue to increase with lower slope after menopause. Three main factors which might cause this difference are younger age, probable more ERN tumors and different histopathology of breast cancer patients in Iran, neighboring and other similar countries in comparison to the US and other western countries. More future studies showing definite causes of difference may help in improving prevention, diagnosis and treatment decisions.

## Notes

***Source of funding:*** Gynecology Translational Research Center.
